# Age-, Gender-, and *in Vivo* Different Doses of Isoproterenol Modify *in Vitro* Aortic Vasoreactivity and Circulating VCAM-1

**DOI:** 10.3389/fphys.2018.00020

**Published:** 2018-01-24

**Authors:** Betzabé Nieto-Lima, Agustina Cano-Martínez, María E. Rubio-Ruiz, Israel Pérez-Torres, Verónica Guarner-Lans

**Affiliations:** ^1^Department of Physiology, Instituto Nacional de Cardiología “Ignacio Chávez”, Mexico City, Mexico; ^2^Department of Pathology, Instituto Nacional de Cardiología “Ignacio Chávez”, Mexico City, Mexico

**Keywords:** gender, age, inflammatory cytokine, VCAM-1, isoproterenol, aortic vasoreactivity, NE-induced contraction, Ach-induced vasorelaxation

## Abstract

Different human-like cardiomyopathies associated to β-adrenergic stimulation are experimentally modeled in animals through variations in dose, route, and duration of administration of different cardiotoxic drugs. However, associated changes in the vasculature and their relation to systemic inflammation, and the influence of cardiovascular diseases risk factors (gender and age) upon them are seldom analyzed. Here we studied the effect of age and gender on the vasoreactivity of aortas from mice subjected to *in vivo* repeated β-adrenergic stimulation with different doses of isoproterenol (ISO) in association with circulating inflammatory cytokines. Young (2 months) and old (18 months) male and female mice received 0 (control), 5, 40, 80 or 160 μg/g/d of ISO (7 days, s.c.). IL-1α, IL-4 and vascular cell adhesion molecule-1 (VCAM-1) were quantified in plasma. *In vitro*, norepinephrine-induced vasoconstriction and acetylcholine-induced relaxation were measured in aortas. No differences in contraction, relaxation, IL-1α, and IL-4 were found between control young males and females. Age decreased contraction in males and relaxation was lower in females and abolished in males. VCAM-1 was higher in young males than in females and increased in old mice. Vasoconstriction in ISO-treated mice results as a bell-shaped curve on contraction in young and old males, with lower values in the latter. In females, ISO-160 increased contraction in young females but decreased it in old females. Vasorelaxation was reduced in ISO-treated young males and females. ISO-80 and 160 reduced vasorelaxation in old females, and intermediate doses relaxed aortas from old males. VCAM-1 was higher in young and old males with ISO-80 and 160; while VCAM-1 was higher only with ISO-160 in old females. Our results demonstrate that repeated β-adrenergic stimulation modifies vascular reactivity depending on gender, age, and dose. Females were less sensitive to alterations in vasoreactivity, and young females required a higher amount of the adrenergic stimuli than old females to show vascular alterations. Changes were independent of IL-1α and IL-4. VCAM-1 only changed in old females stimulated with ISO 160. Our results highlight the relevance of considering and comparing in the same study females and aged organisms to improve the accuracy of applications to clinical studies.

## Introduction

β-adrenoceptors (β-AR) are essential regulators of the cardiovascular homeostasis. They are located in the heart, but also in vascular smooth muscle cells, where they mediate vasodilating effects of endogenous catecholamines (Chruscinski et al., 2001). Their overstimulation with catecholamines is associated with heart damage and eventually heart failure (Grimm et al., 1998; Osadchii, 2007; Shao et al., 2013). Heart damage and failure decrease cardiac output and induce changes in vascular function leading to increased systemic resistance to compensate for the decreased output, thus helping to maintain perfusion to vital organs (Ledoux et al., 2003). In this paper, we study the effect of repeated daily β-adrenergic overstimulation with isoproterenol (ISO), a non-selective β-adrenergic agonist on aortic vascular reactivity.

β-adrenergic stimulation also increases circulating inflammatory cells (Mills et al., 2002; Barnes et al., 2015), elevating inflammatory mediators including vascular cell adhesion molecule-1 (VCAM-1) and tumor necrosis factor alpha (Chen et al., 2005; Han et al., 2016). Systemic inflammatory cytokines such as interleukin-1 alpha (IL-1α) are also increased when there is damage to organs including the cardiac tissue (Sprague and Khalil, 2009). In addition, the damaged heart is infiltrated with eosinophils that secrete interleukin-4 (IL-4) (Diny et al., 2017). Changes in circulating inflammatory mediators and cell adhesion molecules influence vascular function (Sprague and Khalil, 2009). Particularly VCAM-1 promotes the recruitment of inflammatory cells and other inflammatory cytokines (Cook-Mills et al., 2011). This inflammatory environment in the vascular tissue can trigger remodeling (Ganss et al., 2002; Meloche et al., 2017), cause stiffness of the aorta (Tomiyama et al., 2017) and produce vascular dysfunction, thus modifying the vascular compensatory function.

Experimentally, different pathological cardiac phenotypes result from the stimulation with ISO depending on the dose, route or duration of the administration. These phenotypes resemble different human-like cardiomyopathies including myocardial infarction (Hohimer et al., 2005; George et al., 2010), heart failure (Grimm et al., 1998), cardiac hypertrophy (Ma et al., 2011) and cardiomyopathies induced by stress such as takotsubo syndrome (Shao et al., 2013). However, changes in vascular function after the stimulation of the β-AR are seldom explored, and circulatory markers of inflammation are not usually measured (Davel et al., 2008; Han et al., 2016).

Furthermore, factors such as gender and age influence the incidence of cardiovascular diseases. Aging increases 10-fold the risk of cardiovascular morbidity between ages 50 and 80 (Ghebre et al., 2016). Prevalence of coronary heart disease is higher in men in all age ranges until after 75 years of age (Mosca et al., 2011). However, in women, relative mortality risk linked with cardiac hypertrophy which is the most potent cardiovascular risk factor after age (Levy et al., 1990) is higher (Deo et al., 2011). Aging is also accompanied by increases in inflammation and oxidative stress (El Assar et al., 2013; Wu et al., 2014), by decreases in the contractile responses of vascular smooth muscle and only in certain animal species by elevations in blood pressure (Harvey et al., 2015). The ability of β-AR to respond to catecholamine stimulation declines with age (Sato et al., 1995; Ferrara et al., 2014). Intact blood vessels from female murine models produce or release more endothelial-derived releasing factors such as nitric oxide and less endothelial-derived contracting factors (Kauser and Rubanyi, 1995). Also, the impaired endothelium-dependent aortic relaxation in old male mice is due to enhanced superoxide production via NADPH oxidase, while the relative preservation of endothelial function in female-old aortas may be due to enhanced superoxide scavenging (Takenouchi et al., 2009). Although gender and age play an important role in determining the incidence of cardiovascular diseases (Bhupathy et al., 2010), female and old organisms are less frequently considered as targets for the study of diseases, toxicity, drugs, and therapies.

Here we studied the differences in *in vitro* norepinephrine (NE)-induced contraction and acetylcholine (Ach)-induced vasorelaxation in young and old male and female mice, previously injected *in vivo* with different repeated doses of ISO. This study is important since sensitivity to heart damage caused by the stimulation with different doses of catecholamines that act on the β-adrenergic system is modulated by risk factors such as age and gender. In addition, there is little information on the relation of β-adrenergic stimulation, vasculature function and inflammation which potentiates damage, and therefore, our aim was also to measure the circulating inflammatory mediators IL-1α, IL-4, and VCAM-1 which are known to influence vascular function.

## Materials and methods

### Animals and *in vivo* treatment with isoproterenol

All procedures followed the guidelines established by the Federal Regulation for Experimentation and Animal Care (SAGARPA, NOM-062-ZOO-1999, México) and the experimental protocol was registered and approved by our institution; protocol INCICH-10-695. Young (2 months old) and old (18 months old) male and female Balb/c mice were used. Animals were kept with food and water *ad libitum* and under a 12:12 light:dark cycle. Before treatment began, animals were inspected to ensure their good health. For each age and gender, five groups of four mice each were formed. Each group received subcutaneously one of the following doses of ISO for seven days: 5, 40, 80 and 160 μg/g/d. These doses were chosen considering reported minimal and maximal ranges that induce heart damage but not the death of the organisms (Wallner et al., 2016). The administration for 7 days was chosen since we reported that it produces damaging effects on the heart (Nieto-Lima et al., 2013, 2016). One group of each age and gender received saline solution as a vehicle. 24 h after the last administration, mice were weighed, sacrificed and blood was collected by heart puncture in syringes with EDTA. Plasma was obtained by centrifuging the blood at 2500 g for 10 min at 4°C. Ventricles and aortas were removed. Ventricles were washed in phosphate buffer solution and weighed. The ventricular weight (VW) to body weight (BW) ratio (VW/BW) was used as a macroscopic evidence of heart damage. Aortas were immediately placed in oxygenated normal tyrode solution (containing in mM: 140 NaCl, 5 KCl, 1 CaCl_2_, 1 MgCl_2_, 5 HEPES, and 5.5 glucose; pH 7.4) and used to determine *in vitro* contraction to NE and relaxation to Ach.

### *In vitro* NE-induced vasoconstriction and Ach-induced vasorelaxation in aortas from vehicle- and isoproterenol-treated young and old male and female mice

Aortas from 4 mice of each group were obtained and carefully cleaned from connective and adipose tissue, taking care not to damage the endothelium. 1 or 2 segments from every aorta were used totaling 6–8 vascular reactivity assays. Tension measurements were made as previously described (Rubio-Ruiz et al., 2014). Briefly, a 1.5 g resting tension was applied to aortic rings (segments of about 3–4 mm long). This tension has been tested previously and found to be optimal under our experimental conditions. The aortas were allowed to rest for 60 min, with the replacement of the tyrode solution every 20 min. As in most studies of vascular reactivity (Kamata et al., 1989; Baños et al., 1997; Ponnoth et al., 2008; Rubio-Ruiz et al., 2014), the aortas were stimulated twice with NE (1 μmol/L). Endothelial integrity was tested by Ach induced relaxation (10 μmol/L) (Furchgott et al., 1984) in pre-contracted aortas with NE (1 μmol/L). Vasorelaxation was determined by cumulative concentration-response curves to Ach (10^−4^–10^−9^ M) on NE- (1 μmol/L) precontracted aortic rings. The half maximal response to Ach (pEC_50_), expressed as –log10 of the molar concentration of EC_50_, and the maximum relaxation response (E_max_) were calculated. To have an approach on the participation of nitric oxide (NO) on the vasorelaxation, we measured nitrates (${\text{NO}}_{3}^{-}$) and nitrites (${\text{NO}}_{2}^{-}$), as previously reported (Pérez-Torres et al., 2014). NO is a relaxing factor synthesized not only from L-arginine by NO synthases (NOSs) but also from its inert metabolites, the nitrites and nitrates. ${\text{NO}}_{3}^{-}$ was reduced to ${\text{NO}}_{2}^{-}$ by nitrate reductase enzyme reaction. Ten μl of serum were added to 5 μl nitrate reductase (0.020 units) and 30 μl of buffer (0.14 M KHPO_4_, pH 7.35) and incubated for 30 min at 37°C. At the end of the incubation period, 50 μl of sulfanilamide 1% and 50 μl of N-naphthyl-ethyldiamine 0.1% were added, and the total volume was adjusted to 1 ml. The calibration curve was obtained with a solution of KNO_2_ ranging 5–0.078 M. The absorbance was measured at 540 nm.

### Inflammatory profile quantification

Plasma circulating levels of IL-1α (RRA00, R&D Systems), IL-4 (R4000, R&D Systems), and VCAM-1 (E-EL-R1061, Elabscience) were determined by enzyme-linked immunosorbent assay (ELISA) following the manufacturer's instructions using 10 μl of serum from all experimental animal groups (*n* = 4 per group).

### Statistical analysis

Statistical analysis of vascular reactivity assays, pEC_50_ and E_max_ were performed by two-way ANOVA, followed by Student-Newman-Keuls or Dunn tests, using the Sigma Stat program (Jandel Scientific). When comparing control values between gender and age, Student's *t*-test was used with the same program. Statistical analysis of plasma interleukins was made using Student's *t*-test with the Sigma Stat program (Jandel Scientific). Statistical analysis of body weight was performed by two-way ANOVA, followed by Sidak test, and VW/BW was performed by one-way ANOVA, followed by Dunnet test, using Prisma software. Results are expressed as the mean ± standard error of the mean (SEM). Differences were considered statistically significant when *p* < 0.05.

## Results

### *In vitro* NE-induced vasoconstriction and Ach-induced vasorelaxation of aortas from control young and old male and female mice

The contraction induced by NE was slightly stronger in aortas from males than from females of 2 months of age (0.21 ± 0.02 g vs. 0.18 ± 0.03 g, respectively) but the difference was not statistically significant. In aged mice, contraction significantly decreased in male aortas, while it remained constant in females (old males: 0.06 ± 6.8^*^10^−3^g vs. old females: 0.17 ± 0.01 g, respectively).

Aortic rings exhibited a concentration-dependent vasorelaxation in response to Ach. The maximal relaxation was similar in aortas from male and female mice of 2 months of age (E_max_ = 76.7 ± 0.9% vs. 71.6 ± 0.4%, respectively) (Table 1). Vasorelaxation significantly decreased in aged female aortas (E_max_ = 71.6 ± 0.4% vs. 50.0 ± 2.2%); and in male aortas, vasorelaxation was abolished (Figures 1A,B). The EC_50_ was not altered in any group (Table 1). There was a clear tendency of ${\text{NO}}_{3}^{-}$ and ${\text{NO}}_{2}^{-}$ to decrease with age which was not statistically significant in both genders (males: young 20.53 ± 4.07 nM/ml vs. old 12.82 ± 4.22; females: young 10.22 ± 4.86 vs. old 7.25 ± 2.13 nM/ml).

**Table 1:** *In vitro* sensitivity to Ach and maximum relaxation response of aortas from young (2 months) and old (18 months) male and female mice treated with isoproterenol.

	**Young (2 months old)**		**Old (18 months old)**	
	**pEC_50_**	**Emax (%)**	**pEC_50_**	**Emax (%)**
**MALES**				
Vehicle	6.24 ± 0.03	76.7 ± 0.9	ND	ND
ISO 5 μg/g/d	6.13 ± 0.13	49.7 ± 2.7^*^	6.01 ± 0.02	42.5 ± 0.4^*^
ISO 40 μg/g/d	6.45 ± 0.23	31.3 ± 2.9^*^	5.47 ± 0.07	40.4 ± 1.7^*^^#^
ISO 80 μg/g/d	5.94 ± 0.16	39.4 ± 1.9^*^	6.02 ± 0.05	50.9 ± 1.3^*^^#^
ISO 160 μg/g/d	5.88 ± 0.21	34.3 ± 3.5^*^	ND	ND
**FEMALES**				
Vehicle	6.02 ± 0.01	71.6 ± 0.4	6.29 ± 0.10	50.0 ± 2.2^&^^#^
ISO 5 μg/g/d	5.54 ± 0.10^*^	50.4 ± 2.8^*^	6.13 ± 0.20^#^	33.4 ± 2.8^*^^&^^#^
ISO 40 μg/g/d	6.47 ± 0.15	49.9 ± 3.0^*^^&^	6.23 ± 0.13^&^	40.1 ± 2.6^*^^#^
ISO 80 μg/g/d	6.18 ± 0.12	49.8 ± 2.6^*^	5.94 ± 0.07	28.0 ± 1.2^*^^&^^#^
ISO 160 μg/g/d	5.82 ± 0.16	48.2 ± 3.9^*^^&^	6.02 ± 0.12	20.6 ± 1.2^*^^&^^#^

*Values are means ± SEM, n = 6–8 vasoreactivity assays. Aortas were pre-constricted with NE 1 μM. ISO doses were 5, 40, 80, and 160 μg/g/d for 7 days. pEC_50_ (–log_10_ de EC_50_), sensitivity to Ach; E_max_, maximum relaxation response; ISO, isoproterenol; ND, not detected*.

* p < 0.05 vs. vehicle same group;

& p < 0.05 vs. male same age and dose;

# *p < 0.05 vs. young same sex and dose determined by Student's t-test (controls) and two-way ANOVA and post hoc tests*.

**Figure 1:**
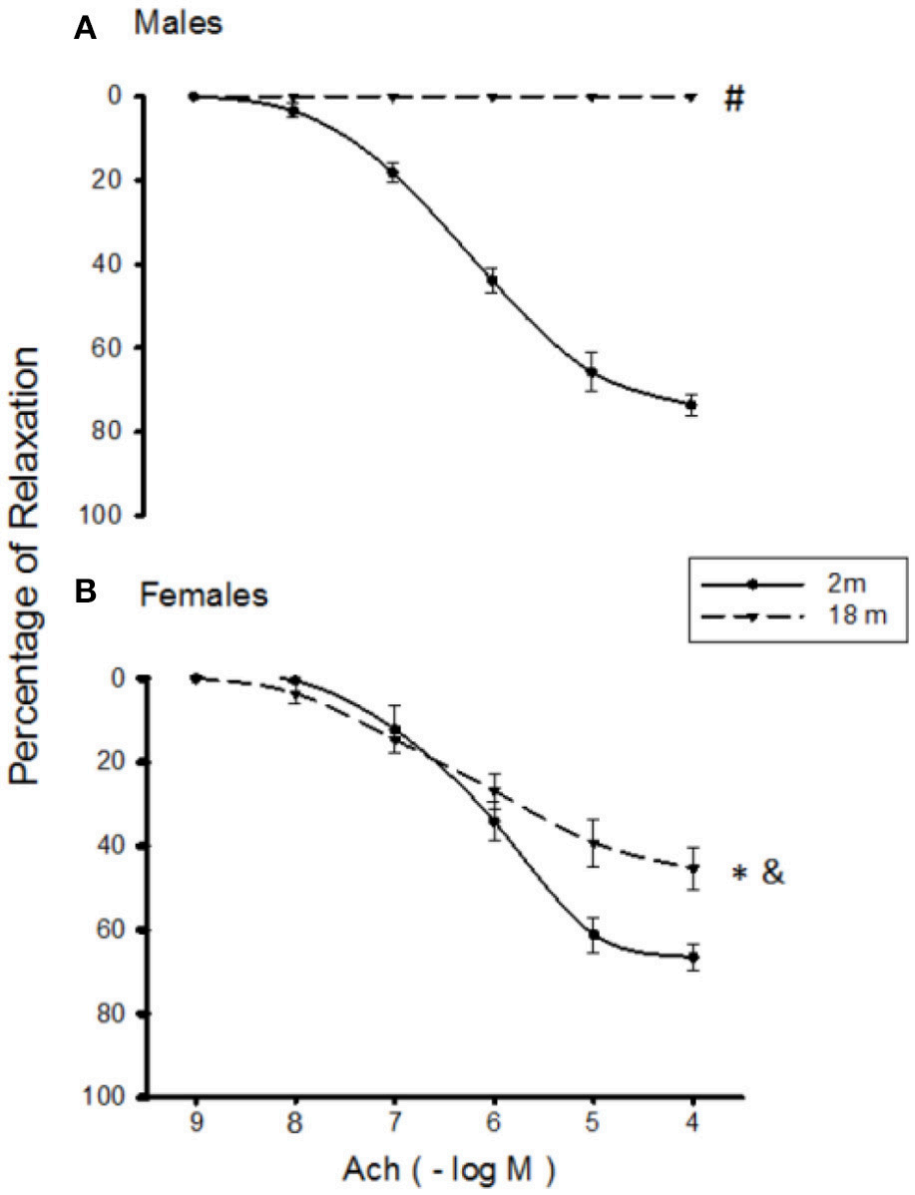
Ach-induced relaxation in NE-precontracted aortic rings from 2 months-old (continuous line) and 18 months-old (discontinuous line) male **(A)** and female **(B)** mice. Values are means ± SEM; *n* = 6–8 vasoreactivity assays. ^#^*p* < 0.01 old males vs. young males; ^*^p < 0.05 old females vs. young females; ^&^*p* < 0.01 old females vs. old males determined by Student's *t*-test.

### *In vitro* NE-induced vasoconstriction and Ach-induced vasorelaxation of aortas from isoproterenol-treated young and old male and female mice

**Figure 2:**
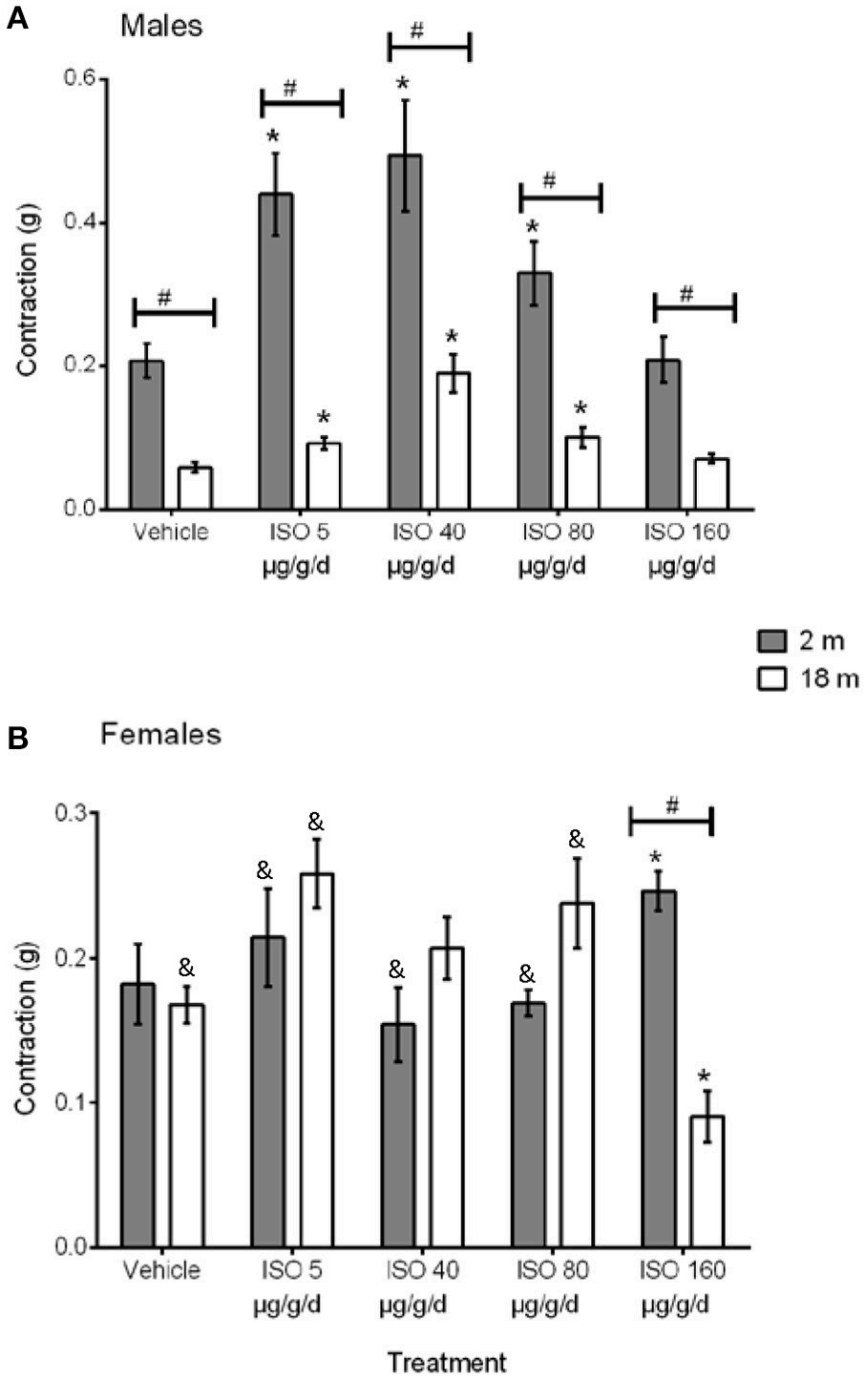
NE-induced contraction in 2 months-old (solid bars) and 18 months-old (open bars) male **(A)** and female **(B)** aortas from vehicle- and ISO-treated mice. Values are means ± SEM; *n* = 6–8 vasoreactivity assays. ISO doses were 5, 40, 80, and 160 μg/g/d for 7 days. ISO, isoproterenol. ^*^p < 0.05 vs. vehicle same group; ^&^*p* < 0.05 vs. male same age and dose; ^#^*p* < 0.05 vs. young same sex and dose determined by two-way ANOVA and post hoc tests.

*In vivo* treatment with different doses of ISO produced a bell-shaped curve on the NE-induced contraction in aortas from young male mice (Figure 2A); while in young females, only the highest dose of ISO significantly increased vascular contraction (Figure 2B). The contraction in old male mice showed the same tendency as aortas from young male mice but with a reduced contraction force (Figure 2A). In contrast, in aged female mice, only the *in vivo* treatment with the highest dose of ISO significantly decreased vascular contraction when compared to its control and young females for the same dose (Figure 2B).

**Figure 3:**
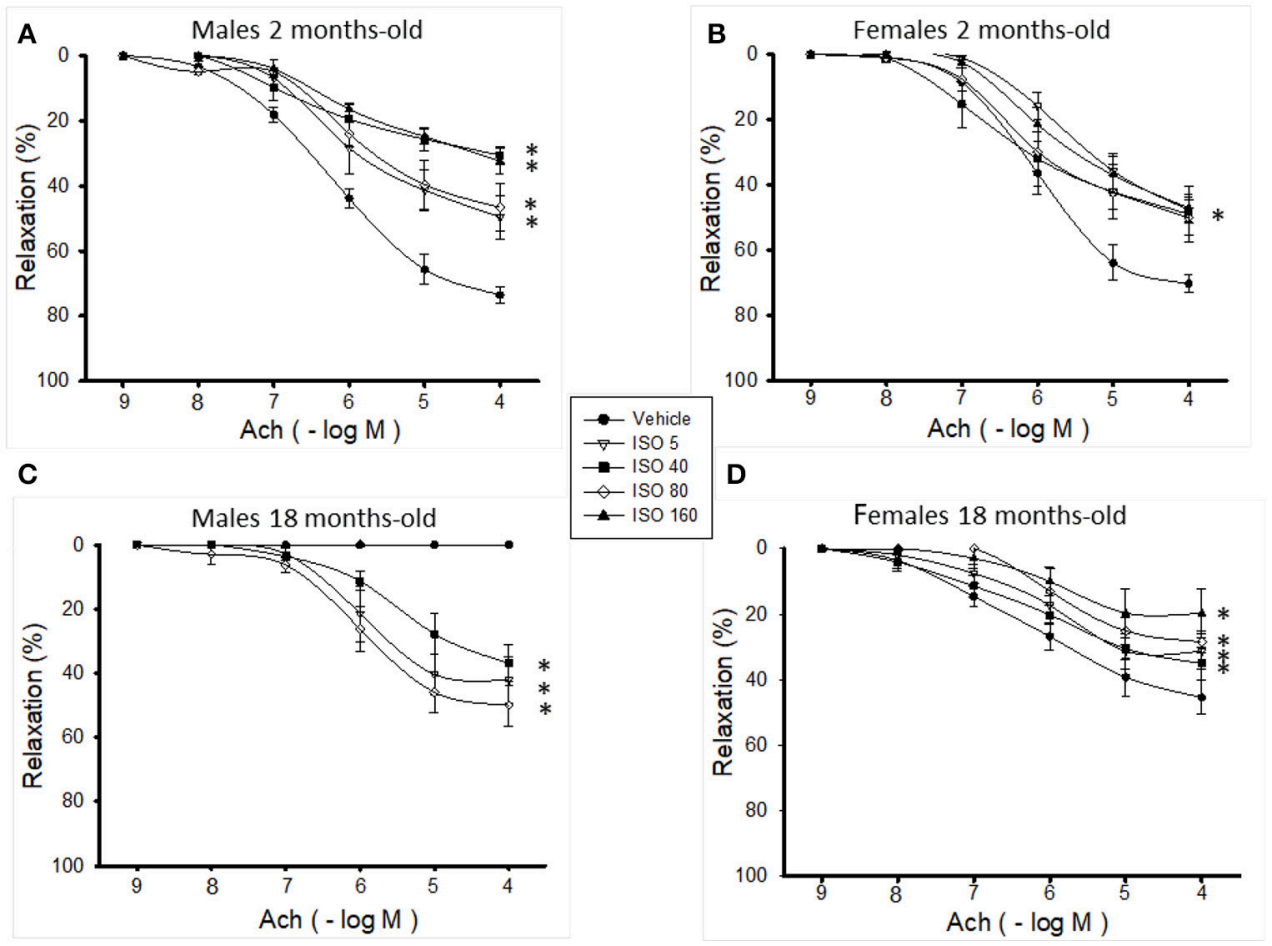
Effect of ISO-treatment on vascular relaxation in aortic rings from 2 months-old male mice **(A)**, 2 months-old female mice **(B)**, 18 months-old male mice **(C)**, and 18 months-old female mice **(D)**. Values are means ± SEM; *n* = 6–8 vasoreactivity assays. ISO doses were 5, 40, 80, and 160 μg/g/d for 7 days. ISO, isoproterenol. ^*^p < 0.05 vs. vehicle same group determined by two-way ANOVA and *post hoc* tests.

As shown in Figures 3A,B, ISO treatment significantly decreased vascular relaxation of aortic rings from young male and female mice in a non-dose-dependent manner; however, the decrease in the maximum relaxation response was more pronounced in males (50 vs. 30%, respectively; Table 1). ISO treatment in young male mice did not affect the sensitivity to Ach, while in young females, the dose of 5 μg/g/d significantly diminished the response to Ach, as observed from the increased value of pEC_50_ (Table 1). *In vivo* treatment with 5, 40, and 80 μg/g/d of ISO relaxed the aortas from old male mice, and there was no relaxation with the highest dose (Figure 3C and Table 1). In old female mice, ISO significantly reduced the relaxation (in approximately 50%) with all of the doses (Figure 3D and Table 1). There were no significant changes in ${\text{NO}}_{3}^{-}$ and ${\text{NO}}_{2}^{-}$ concentration with any of the doses of ISO used in young or aged males and females.

### Plasma concentration of IL-1α and IL-4 and circulating VCAM-1 in control and isoproterenol-treated mice

**Figure 4:**
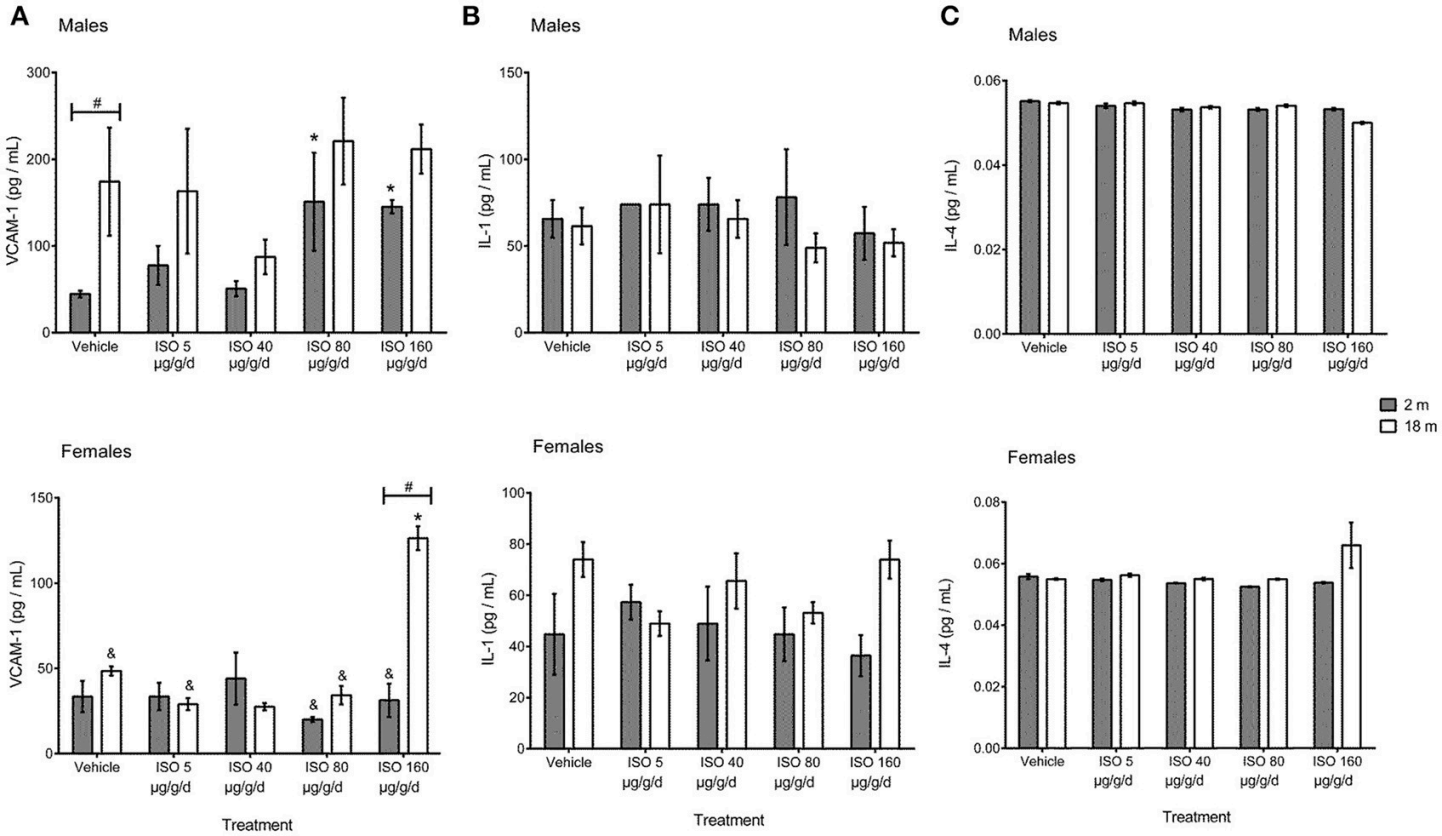
Plasma concentrations of circulating inflammatory mediators VCAM-1 **(A)**, IL-1α **(B)**, and IL-4 **(C)** from young (2 months) and old (18 months) male and female mice treated with isoproterenol. Values are means ± SEM, *n* = 4 mice. ISO doses were 5, 40, 80, and 160 μg/g/d for 7 days. ISO, isoproterenol; IL-1α, interleukin-1 alpha; IL-4, interleukin-4. ^*^p < 0.05 vs. vehicle same group; ^&^*p* < 0.05 vs. male same age and dose; ^#^*p* < 0.05 vs. young same sex and dose determined by Student's *t*-test.

Changes in plasma concentrations of circulating VCAM-1, IL-1α, and IL-4 are shown in Figure 4. Plasma concentrations of IL-1α and IL-4 were not different between vehicle-treated males and females and between vehicle-treated young and old mice. VCAM-1 was significantly higher in control males than females (44.5 ± 3.9 vs. 33.5 ± 9.1 pg/mL), and levels were increased with age, although they did not reach statistical significance. Old male mice had the highest levels of VCAM-1 when compared to the rest of the groups (by approximately 4.13–fold levels to the rest of the groups).

*In vivo* treatment with ISO did not modify plasma levels of IL-1α and IL-4 in any of the groups. Circulating VCAM-1 was considerably increased in young males with the doses of 80 and 160 μg/g/d (3.39- and 3.26-fold vs. its corresponding vehicle), without changes in old males. Meanwhile, circulating VCAM-1 was not modified by ISO in young females, but it was significantly increased with dose of 160 μg/g/d in old females (2.58-fold vs. control).

### Effect of *in vivo* treatment with isoproterenol on body weight and VW/BW

Changes in ventricular weight (VW) to body weight (BW) ratio (VW/BW) were quantified to corroborate that the doses used induced heart damage. Therefore, the observed changes in vasoreactivity can be related to cardiac damage. Table 2 shows changes in body weight (BW) and ventricular weight to body weight index (VW/BW) in young and old male and female mice treated with vehicle or ISO. BW of vehicle-treated male mice was significantly higher than that of females for both ages (young: 26.65 ± 0.55 g vs. 21.95 ± 0.52 g; old: 30.86 ± 0.71 g vs. 26.93 ± 0.66 g, respectively). Old mice had a higher weight than young mice in both genders (*p* < 0.05). VW/BW was higher in vehicle-treated males than in females in both ages (young: 4.7 ± 0.06 vs. 4.4 ± 0.08, old: 5.0 ± 0.07 vs. 4.1 ± 0.12, respectively) but only reached statistical significance in vehicle-treated old mice. Treatment with ISO did not modify the gender and age-associated BW, but it significantly increased VW/BW in all groups.

**Table 2:** Body weight and ventricular weight/body weight ratio from young (2 months) and old (18 months) male and female mice treated with isoproterenol.

	**Young (2 months old)**			**Old (18 months old)**		
	**Initial BW (g)**	**Final BW (g)**	**VW/BW ratio**	**Initial BW (g)**	**Final BW (g)**	**VW/BW ratio**
**MALES**						
Vehicle	26.32 ± 0.65	26.65 ± 0.55	4.7 ± 0.06	31.06 ± 1.15^&^	30.86 ± 0.71^&^	5.0 ± 0.07
ISO 5 μg/g/d	26.06 ± 0.74	26.53 ± 0.66	5.2 ± 0.09^#^	30.77 ± 0.98^&^	30.00 ± 0.70^&^	6.0 ± 0.13^#^
ISO 40 μg/g/d	26.07 ± 0.48	25.90 ± 0.67	5.8 ± 0.06^#^	34.09 ± 1.23^&^	33.36 ± 1.02^&^	5.7 ± 0.07^#^
ISO 80 μg/g/d	26.79 ± 0.65	26.60 ± 0.72	5.8 ± 0.09^#^	32.15 ± 0.91^&^	30.35 ± 0.93^&^	5.8 ± 0.07^#^
ISO 160 μg/g/d	27.42 ± 0.89	26.92 ± 1.03	5.9 ± 0.10^#^	32.25 ± 0.74^&^	30.47 ± 0.67^&^	6.0 ± 0.12^#^
**FEMALES**						
Vehicle	21.94 ± 0.44^*^	21.95 ± 0.52^*^	4.4 ± 0.08	27.79 ± 0.57^&^	26.93 ± 0.66^*^^&^	4.1 ± 0.12^*^
ISO 5 μg/g/d	21.91 ± 0.73^*^	22.38 ± 0.85^*^	5.5 ± 0.07^#^	27.58 ± 0.49^&^	27.30 ± 0.48^&^	4.7 ± 0.08^#^
ISO 40 μg/g/d	20.83 ± 0.90^*^	21.61 ± 1.00^*^	5.6 ± 0.11^#^	27.69 ± 1.08^*^^&^	25.36 ± 0.82^*^^&^	5.1 ± 0.09^#^
ISO 80 μg/g/d	20.97 ± 1.00^*^	21.62 ± 0.88^*^	5.7 ± 0.11^#^	28.54 ± 1.01^*^^&^	26.59 ± 1.14^*^^&^	5.3 ± 0.14^#^
ISO 160 μg/g/d	21.44 ± 0.56^*^	21.21 ± 0.72^*^	6.1 ± 0.07^#^	28.70 ± 1.11^&^	26.52 ± 0.88^*^^&^	5.4 ± 0.08^#^

*Values are means ± SEM, n = 4 mice. ISO doses were 5, 40, 80 and 160 μg/g/d for 7 days. BW, body weight; VW, ventricular weight; ISO, isoproterenol*.

# p < 0.05 vs. vehicle same group;

* p < 0.05 vs. male same age and dose;

& *p < 0.05 vs. young same sex and dose determined by one- (VW/BW ratio) and two-way ANOVA (BW) and post hoc tests*.

## Discussion

Overstimulation of the β-adrenergic system with daily doses of ISO causes heart damage inducing inflammation of the tissue and having consequences on the vasculature. In this paper, we studied the effects of repeated β-adrenergic overstimulation with different doses of ISO on vascular reactivity.

We found that repeated β-adrenergic stimulation with different doses of ISO modifies vascular reactivity (VR) in an age-, gender-, and dose-dependent pattern being young males the most susceptible to vascular alterations; whereas old mice tended to keep their homeostasis, but with different strategies according to the gender. Even though changes in vascular tone may be associated with systemic inflammation induced by the repeated β-adrenergic stimulation, in our study, we found no changes in the circulating levels of the inflammatory mediators IL-1α and IL-4 and only VCAM-1 seemed to be associated with vascular alterations in old females stimulated with the highest ISO dose. However, we cannot rule out that other biochemical and cellular markers of inflammation and local inflammation markers could be modified.

### Effect of gender and age on vascular reactivity and inflammation

Gender and age modulate the incidence and progression of vascular and heart-related diseases. In the present study, we found no differences in VR between vehicle-treated young males and females. This response is in agreement with previous reports (Júnior and Cordellini, 2007; Loria et al., 2014).

Vasoconstriction usually declines with age due to decreases in cell density, aortic compliance and aortic contractility and increases in aortic diameter, the thickness of the medial layer of the aortic wall and collagen volume fraction (Wheeler et al., 2015). The decrease in endothelin-1 secretion could also decrease contraction (Leblanc et al., 2013) during aging. Our results corroborate that vascular contraction decreases with age but only in males. Similar results in aged animals have been previously reported (Gurdal et al., 1995; Shipley and Muller-Delp, 2005; Blough et al., 2007; Wheeler et al., 2015).

Gender differences in contraction during aging may be related to sex hormones. Estrogens improve endothelial function (Usselman et al., 2016), and inflammation (Zhu et al., 2013) throughout life. They lower levels of oxidative stress (Zhu et al., 2013; Angeloni et al., 2017). This decrease may translate into less damage to the structure and function of the vasculature and the heart. Testosterone may also be involved, since postmenopausal women with chronic heart failure are benefited by testosterone supplementation and hypertensive men have lower serum testosterone levels than normotensive men of the same age (Lopez-Ruiz et al., 2008).

Endothelium-dependent relaxation mediated by Ach has been reported to fall during rat maturation (Soltis and Newman, 1992). Our study corroborates that relaxation decreases with age and this response is more pronounced in males. One of the mediators of vasorelaxation is NO, and plasma nitrite serves as a reservoir of NO bioactivity (Cao et al., 2009; Waltz et al., 2015). Here we found a tendency of reduced levels of ${\text{NO}}_{3}^{-}$ and ${\text{NO}}_{2}^{-}$ during aging in males and females. However, we cannot exclude the participation of endothelium in relaxation because nitrates and nitrates were measured in plasma, which does not allow us to ensure that its source is exclusively the endothelium.

Besides cardiovascular modifications, aging also influences the inflammatory system. In the present study, circulating IL-1α and IL-4 were not different among individuals from different gender and age, but young vehicle-treated males had higher levels of circulating VCAM-1. At the vascular level, previous studies indicate that circulating pro-inflammatory factors may activate endothelial cells to promote an atherogenic phenotype, which may result in endothelial dysfunction, ventricular dysfunction and heart failure (Marchais et al., 1993; Belz, 1995; Ganss et al., 2002; Safar et al., 2012; Meloche et al., 2017; Tomiyama et al., 2017). Endothelium-independent regulation might also participate since the involvement of the endothelium was not evidenced in our experiments. In accordance with the participation of VCAM-1 in the alteration of the heart and vascular function, increased levels of this molecule have been correlated with the development of cardiovascular diseases in healthy middle-aged men during a 6.6 years follow-up study (Schmidt et al., 2009). Even more, VCAM-1 has been proposed as a reference molecule for diagnosis of viral myocarditis (Gao et al., 2013) and is used to identify subjects at risk for events related to heart failure (Savic-Radojevic et al., 2013). Therefore, higher levels of VCAM-1 may contribute to a faster or sooner decline in male mice health, at least when compared to females. In line with this, we found that old males showed the highest value of VW/BW and VCAM-1 and an altered VR (reduced vasoconstriction and non-existent vasorelaxation with Ach). In contrast, old females only showed a slight reduction in vasorelaxation without significant changes in VW/BW, vasoconstriction, and VCAM-1.

### Effect of isoproterenol on VR and inflammation in young and aged male and female mice

In the present work, the repeated stimulation of the β-AR system with different doses of ISO that caused heart damage also triggered different responses in VR and inflammation according to the gender and age of the organisms.

We found that, in response to β-adrenergic stimulation, young male aortas showed a bell-shaped contraction curve with increases in 3 of 4 doses of ISO and this response was decreased with age. In young female aortas, only the highest dose of isoproterenol produced a significant increase in contraction. In old females, a decrease in contraction was registered only with the highest dose of ISO. Gender differences in the susceptibility to β-adrenergic stimulation have been previously reported, being males more susceptible (Page et al., 2008; Elmes et al., 2009; Michel et al., 2017). In agreement with this, our results show that in young females the dose of catecholamines needed to trigger a vasoreactivity response is higher than in males. Thus, young females are more resistant to vascular responses triggered by β-adrenergic stimulation. Gender-related differences in VR have been previously reported in murine models of diabetes and hypertension (Robert et al., 2005; Takenouchi et al., 2010). These studies also showed that diseases modify the vasorelaxing response in females. There are also changes in VR of aortas of rats during pregnancy that are dependent on the gestational age (Jain et al., 1998); providing evidence of the effect of gender on VR under pathological conditions.

The β2-AR/Giα signaling pathway (Davel et al., 2014), and endothelial nitric synthase uncoupling mediated by oxidative stress (Davel et al., 2014), might participate in the increases in aortic contraction in young males by elevating ROS, generation and impairing NO bioavailability (Davel et al., 2006, 2008). Opposite to males, estrogens in females may protect them from alterations in contraction in most of the doses of ISO used. Estrogens reduce oxidative stress and improve endothelial function (Baylis, 2009). Under pro-inflammatory conditions, estrogens inhibit vasoconstrictor prostanoid production in endothelial cells and activity in intact arteries through G protein-coupled estrogen receptor (GPER) (Meyer et al., 2015).

In old males, a reduced aortic response is expected since the ability of β-AR to respond to the stimulation by catecholamines is declined. In females, the selective blockage of β2-AR increases the contraction to NE (Al-Gburi et al., 2017). Another interpretation might come from the activation of systems such as the renin-angiotensin system which is also influenced by age and gender (Costa et al., 2016).

In our study, ISO-administration was accompanied by a decrease in relaxation in young males and females at all doses. However, we did not find significant changes in the ${\text{NO}}_{3}^{-}$ and ${\text{NO}}_{2}^{-}$ levels with the ISO treatment with age nor with gender. It has been reported that decreases in β-adrenoceptor density (Kiuchi et al., 1993; Gaballa et al., 2001) play an important role in the β-adrenoceptor-mediated reduction in vasorelaxation in animal models of heart failure (Mathew et al., 1993; Nasa et al., 1996; McGoldrick et al., 2007). Autooxidation of catecholamines produces hydrogen peroxide and superoxide anion, and exogenous hydrogen peroxide which might cause contractions in aortas from normotensive rats (Rodríguez-Martínez et al., 1998). Even more, superoxide anion degrades NO, an agent that increases the relaxation and suppresses the contraction of the vessels, leading to the formation of peroxynitrite, an oxidant capable of causing tissue damage (Bouloumié et al., 1997). Similar to autooxidation of catecholamines, β-AR overstimulation may induce degradation of NO through endothelial nitric oxide synthase uncoupling (by decreasing the concentration of biopterin), oxidative stress and inflammation (Davel et al., 2008; Xu et al., 2016). Protection by estrogens may attenuate oxidative stress and inflammation (Viña et al., 2013; Lamas et al., 2015), reducing the degradation of NO, and thus attenuating decreases in vasorelaxation. The latter agrees with the lower decrease in relaxation that we observed in young females compared to young males, although this data did not reach statistical significance. Furthermore, human female macrovessels express more dilatory β1- and β3-adrenoreceptors than male vessels. This gender-specific difference is attenuated with aging (Al-Gburi et al., 2017).

In aortas from old female mice, the highest doses of ISO significantly decreased the maximum response to Ach. Surprisingly, the treatment with ISO at 5, 40, and 80 μg/g/d induced vasorelaxation in aortas from old male mice. We do not know the exact reason for this effect, but we hypothesize that ISO could be mediating the vasorelaxant effect by the stimulation of β2-AR that activate the ATP-sensitive potassium channels in vascular smooth muscle (Fauaz et al., 2000).

The combination of the changes in relaxation and contraction that we observed suggests that young females are more resistant than young males to vascular responses triggered by β-adrenergic stimulation. This is reflected by the decreased relaxation with all the doses without changes in contraction with the exception of the 160 dose in females, while in males, relaxation decreased and contraction increased. This last combination is more likely to lead to the occlusion of the vessels. Opposite to young mice, old mice treated with ISO tended to keep the homeostasis of the vascular function with most of the doses of ISO, but probably through different mechanisms. For instance, in our study, contraction and relaxation increased with the majority of the doses in old males; while in old females there were no changes in vasorelaxation and vasoconstriction when they were treated with different doses of isoproterenol. These results highlight the relevance of including males and females young and old when trying to test new treatments.

Even if inflammation and changes in the vascular function have been associated with several conditions that involve the β-adrenergic overactivation (Zeiher et al., 1994; Hadi et al., 2005; Esler et al., 2006; Stapleton et al., 2008; Hong, 2010; Webb et al., 2011; Sandoo et al., 2013; Moxon et al., 2014), our results show no correlation between the inflammatory mediators (IL-1α, IL-4) and VR. Similar results with a no-significant relationship between the chronotropic 25 dose (CD25) of ISO and interleukin 6 or soluble tumor necrosis factor receptor-1 have been previously reported (Euteneuer et al., 2012). However, we found that the levels of VCAM-1 were higher in males than in females and that they increased with age. VCAM-1 is a soluble biomarker of endothelial activation, which promotes a response of the macro- and microvasculature to an injury involving several phenotypic changes of the luminal surface of endothelial cells (Jefferson et al., 2013). These changes are necessary for efficient leukocyte adherence and diapedesis to injured tissues. Aging is associated with an elevated level of a chronic endothelial activation (Belliere et al., 2015) and thus, VCAM-1 is expected to be high in this condition. Importantly, an increase in the levels in VCAM-1 and the lack of detection of other inflammatory cytokines have been reported in the coronary circulation of mice treated with β2-adrenergic blockers. This suggests that β-adrenergic stimulation could regulate VCAM-1 independently of other cytokines (Chen et al., 2005). However, further research on this issue is still needed. In general, our results support the idea that there are differences in male and female responses at different stages of life in association to the repeated overstimulation of the β-AR system.

In conclusion, our results showed that repeated β-adrenergic stimulation with different doses of catecholamines (ISO) modifies VR in an age-, gender- and dose-dependent manner being young males more susceptible to damage. Old mice tend to keep their homeostasis, but with different strategies according to the gender. These alterations in VR accompany the heart damage which was evidenced by the increases in VW/BW. A vicious cycle could be established with a tendency to threaten life unless interrupted. Although inflammation and vascular tone are associated with β-adrenergic stimulation, this association might not necessarily involve the participation of the inflammatory cytokines IL-1α and IL-4. Only circulating VCAM-1 levels showed changes in old females stimulated with the highest ISO dose. Therefore, other cellular and humoral participants of the inflammatory response should also be assayed in future studies. Importantly, these results highlight the relevance of considering the gender and age of organisms in studies aiming to determine mechanisms that lead to the incidence of diseases.

## Author contributions

AC-M and BN-L: designed the study, performed the treatments, obtained the heart and aortic tissues and plasma samples, drafted the interpreted results and wrote and reviewed the manuscript; MR-R: performed the assay of vascular reactivity and drafted the interpreted results; IP-T: performed the assay to determine the inflammatory and nitrite profile; VG-L: was responsible reviewing the manuscript.

### Conflict of interest statement

The authors declare that the research was conducted in the absence of any commercial or financial relationships that could be construed as a potential conflict of interest.
